# Differential Enteric Glial and Mast Cell Characteristics Distinguish Microscopic Colitis Subtypes Relative to Healthy and Disease Controls, with Treatment-Associated Reductions in Glial Reactivity and Mast Cell Degranulation in Collagenous Colitis

**DOI:** 10.3390/cells15191778

**Published:** 2026-09-29

**Authors:** Julie Beaudeau, Celia Escudero-Hernández, Vittorio Abruzzese, Georgios Katinios, Gustav Orell, Nawroz Barazanji, Susanna Walter, Henrik Hjortswang, Vytautas Kiudelis, Juozas Kupčinskas, Andreas Münch, Åsa V. Keita

**Affiliations:** 1 Department of Biomedical and Clinical Sciences, Linköping University, 58185 Linköping, Sweden; julie.beaudeau@liu.se (J.B.); v.abruzz@hotmail.it (V.A.); 2 Institute of Clinical Molecular Biology, Christian-Albrechts-University and University Hospital Schleswig-Holstein, 24105 Kiel, Germany; celia.escudero.hernandez@uva.es; 3 Department of Paediatrics and Immunology, University of Valladolid, 47003 Valladolid, Spain; 4 Department of Gastroenterology and Hepatology, Linköping University Hospital, 58185 Linköping, Sweden; georgios.katinios@regionostergotland.se (G.K.); nawroz.barazanji@liu.se (N.B.); andreas.munch@regionostergotland.se (A.M.); 5 Department of Health, Medicine and Caring Sciences, Linköping University, 58185 Linköping, Sweden; 6 Department of Gastroenterology, Institute for Digestive Research, Lithuanian University of Health Sciences, 50009 Kaunas, Lithuania

**Keywords:** budesonide, collagenous colitis, gastrointestinal pathophysiology, immune cells, inflammation, immune regulation, immunofluorescence, lymphocytic colitis, refractory colitis, RNA-sequencing, tight junctions

## Abstract

**Highlights:**

**What are the main findings?**

- Numbers of enteric glial cells expressing GFAP (EGC^GFAP+^) or S100β (EGC^S100β+^), as well as mast cell numbers and degranulation, distinguish collagenous colitis from lymphocytic colitis and healthy controls.
- EGC^GFAP+^, EGC^S100β+^ and mast cell densities in collagenous colitis resemble those observed in IBS and UC, while increased EGC^GFAP+^ and mast cell degranulation are reduced following budesonide treatment.

**What is the implication of the main finding?**

- Neuroimmune alterations are implicated in microscopic colitis pathophysiology, particularly in collagenous colitis.
- Enteric glial cells may represent potential targets for future mechanistic studies in microscopic colitis.

**Abstract:**

Microscopic colitis (MC) is an inflammatory bowel disease characterized by chronic watery diarrhea. Although barrier dysfunction and immune activation are implicated, underlying mechanisms remain unclear. The aim of this study was to characterize enteric glial cells (EGC) and mast cell distribution across MC subtypes, assess treatment effects, and explore EGC-related gene expression and barrier markers. Colonic biopsies from patients with active collagenous colitis (aCC), budesonide-refractory CC (rCC), and active lymphocytic colitis (LC) were compared to irritable bowel syndrome (IBS), ulcerative colitis (UC), and healthy controls (HC). A subset of CC patients was studied during active disease (aCC) and in clinical remission due to budesonide treatment (tCC). Immunofluorescence quantified EGC positive for glial fibrillary acidic protein (EGC^GFAP+^) and S100 calcium-binding protein B (EGC^S100β+^), mast cell tryptase, and tight junction proteins. RNA-sequencing assessed EGC-related gene expression. Results showed that EGC^GFAP+^ were highest in rCC and decreased following budesonide treatment in aCC, whereas EGC^S100β+^ were highest in aCC, remained unchanged following treatment, resembled IBS, and were reduced compared with UC. Mast cell numbers were higher in aCC than in HC and rCC but remained unchanged following treatment, while mast cell degranulation decreased after treatment. Tight junction proteins were unchanged. RNA-sequencing indicated differential regulation of EGC-related genes. In conclusion, MC shows subtype-specific alterations involving EGC and mast cells, suggesting distinct glial states and differential treatment responsiveness. Our findings support the presence of distinct neuroimmune alterations in CC and LC and highlight EGC as potential contributors to MC pathophysiology.

## 1. Introduction

Microscopic colitis (MC) is an inflammatory bowel disease (IBD) causing chronic non-bloody watery diarrhea that is associated with bloating, abdominal pain, and impaired quality of life [1,2]. MC is most prevalent in middle-aged to elderly individuals and predominantly affects females [3]. Unlike Crohn’s disease (CD) and ulcerative colitis (UC), MC is characterized by a normal-appearing colonic mucosa on endoscopy. Therefore, its diagnosis relies on histopathological evaluation of biopsies [4]. The disease is classified into two primary subtypes: collagenous colitis (CC) and lymphocytic colitis (LC), differentiated by their respective histological features and symptoms [3,5,6]. CC is characterized by colonic epithelial lymphocytosis and the presence of a thickened (≥10 μm) collagen band, whereas LC is characterized by ≥20 lymphocytes in the epithelial layer per 100 epithelial cells without a thickened collagen band [7,8]. Recent transcriptomic and immunological data suggest that CC and LC represent distinct disease entities rather than a single spectrum, with differences in genetic associations, immune activation, and genomic [9] and epithelial pathobiology [10,11]. These also suggest that MC shares similarities with CD and UC. In addition, common symptoms of MC seem to overlap with irritable bowel syndrome (IBS) [12].

Even though the etiology of MC remains incompletely understood, a complex interplay of immune dysregulation and genetic factors seems to be implicated in the pathogenesis of the disease [13]. Intestinal barrier dysfunction, including increased permeability, is a well-recognized feature of gastrointestinal disorders such as CD, UC, and IBS [14,15,16,17,18]. An impaired epithelial barrier function has also been described and presented as contributing to the pathogenesis of MC, with evidence of increased permeability [19], crypt hyperproliferation [11,20], alteration of the transepithelial resistance as well as altered tight junctions [11,21]. Although the inflammatory infiltrate in MC is well described [10], the cellular mechanisms contributing to barrier dysfunction and symptom generation remain incompletely understood [4,5,22].

Emerging evidence from related gastrointestinal disorders suggests an important role for neuroimmune interactions emerging as mediators of gastrointestinal disorders, especially involving enteric glial cells (EGC) [14,16,23,24]. EGC are integral components of the enteric nervous system (ENS), known to regulate intestinal barrier function, modulate immune responses and maintain gut homeostasis [25,26,27]. They constitute a heterogeneous cell population and can be identified using markers such as glial fibrillary acidic protein (GFAP), S100 calcium-binding protein β (S100β), proteolipid protein 1 (PLP1), and Sox10 [28]. Although EGC express different markers, they may reflect distinct phenotypes, functional states, or responses to local microenvironmental cues. Indeed, EGC respond dynamically to microenvironmental changes, including inflammation, by adopting reactive phenotypes that may influence disease processes [28]. Furthermore, EGC and their mediators contribute to intestinal barrier regulation under both physiological and disease conditions [23,29].

Immune-barrier mechanisms have been proposed as contributors to symptom generation in MC [13], but EGC-associated mechanisms have so far, to our knowledge, not been explored. We previously showed a disturbed interaction between EGC and mast cells in the colon of patients with IBS [24], and we recently demonstrated altered levels of EGC and their associated pro-inflammatory proteins in patients with UC during active disease compared to remission and to healthy controls (HC) [30]. Given the overlapping barrier defects and immune features shared between MC, IBS and UC, similar EGC-driven mechanisms may also be involved in MC pathogenesis. The involvement of mast cells has been implicated in MC, with studies demonstrating increased mast cell numbers and degranulation activity compared to HC [10,31], similar to those found in CD and UC, especially in the upper part of the lamina propria [32]. This suggests mast cell contribution to the inflammatory processes.

Today, budesonide is the only effective treatment for MC [33], yet its effects on mucosal immune and EGC populations are largely unexplored [34,35]. A subset of patients with CC exhibits a failing response to budesonide, commonly referred to as refractory CC (rCC), representing a more severe and poorly understood disease phenotype [36]. Transcriptomic studies in CC demonstrate that budesonide dampens inflammatory and epithelial stress responses but fails to fully restore several immune- and barrier-related pathways, particularly in rCC [36]. Based on these observations, we hypothesized that MC would be associated with altered EGC and mast cell characteristics, reflecting subtype-specific neuroimmune mechanisms.

The aim of this study was to map the distribution of EGC and mast cells in patients with CC, rCC, and LC, and to compare these findings with those in HC as well as in patients with UC and IBS, serving as disease controls. The aim was further to analyze the distribution and activation of EGC and mast cells in a subset of patients with active CC (aCC), and after successful budesonide treatment (tCC), as well as to assess EGC and immune signaling-related gene expression by RNA-sequencing. In addition, we aimed to assess the expression of tight junction proteins as a measure of epithelial barrier function in these patients.

## 2. Materials and Methods

### 2.1. Patients and Control Groups

**Figure 1:**
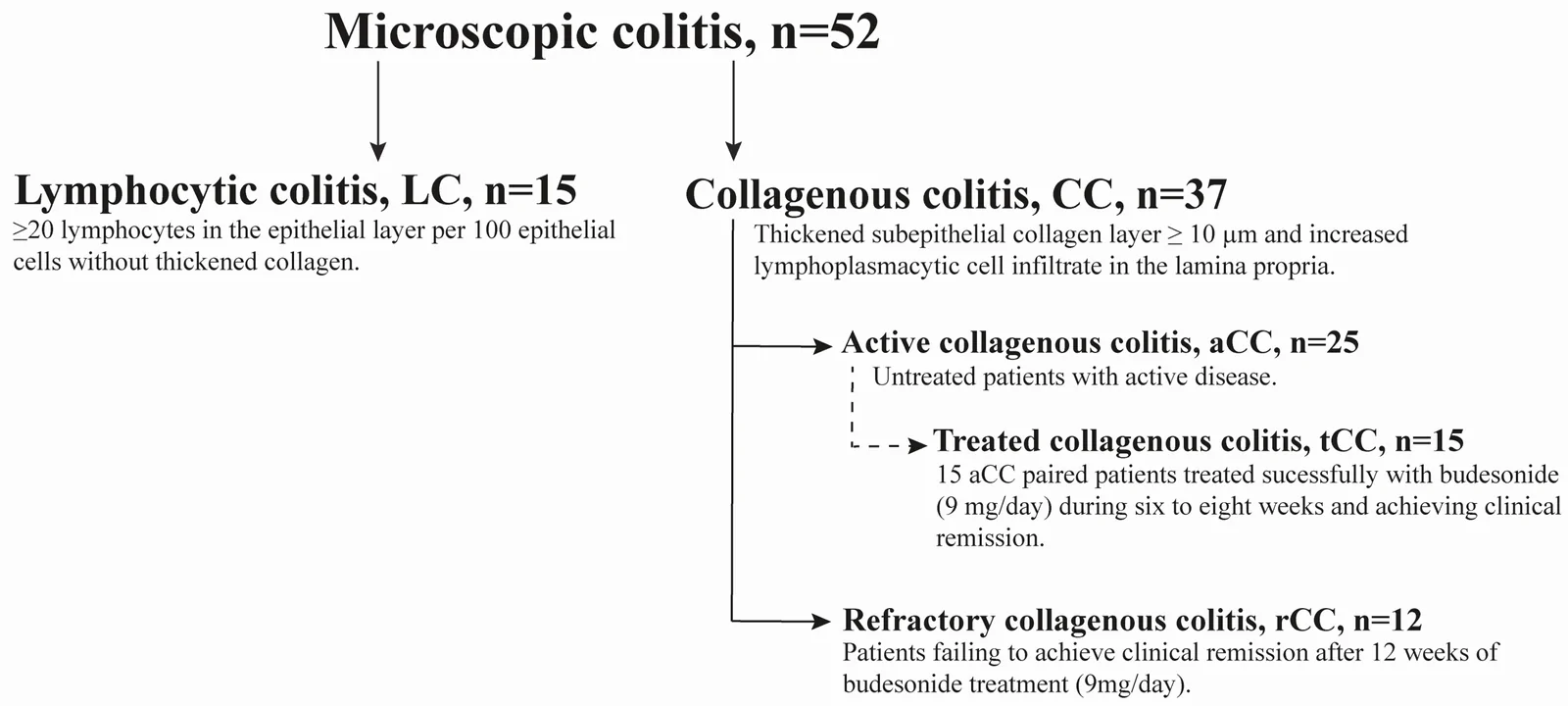
Schematic overview of the different microscopic colitis subgroups included in the study.

Colonic biopsies were collected during sigmoidoscopy from patients with MC at the Department of Gastroenterology and Hepatology at Linköping University Hospital, Linköping, Sweden, and at the Department of Gastroenterology and Institute for Digestive Research at the Lithuania University of Health Sciences, Kaunas, Lithuania. Biopsies were collected from 25 untreated patients with aCC (4 men and 21 women, median age 59 years, range 27–86 years) for comparison with the other MC groups. A subset of 15 of these patients provided paired follow-up biopsies after successful treatment with budesonide (tCC), 9 mg/day, for six to eight weeks (3 men and 12 women, range 27–86 years). These paired samples were analyzed separately, whereas the full aCC cohort was included in the group comparison. Additionally, biopsies from 15 patients with LC (5 men and 10 women, median age 54 years, range 19–80 years) and from 12 patients with budesonide rCC (1 man and 11 women, median age 53.5 years, range 25–75 years) after discontinuation of prolonged treatment due to lack of response (Figure 1). Detailed patient characteristics are shown in Table 1.

As HC, 23 individuals (8 men and 15 women, median age 60 years, range 40–79 years) with no gastrointestinal symptoms or macroscopic/microscopic disease were recruited as a control group. As a first disease control group, biopsies of the inflamed segment from 14 patients (8 men and 6 women, median age 38.5 years, range 21–72 years) with known active UC were included. The extension of the inflammation was noted as distance in cm from the anus and graded according to Endoscopic Mayo score (0–3) [25]. As a second disease control group, biopsies from 26 patients with IBS diarrhea (6 men and 20 women, median age 34.5 years (range 23–51 years) fulfilling ROME IV criteria [37] were included.

### 2.2. Microscopic Colitis Grading and Biopsy Collection

MC was diagnosed according to the European guidelines for MC [33]. Histopathological criteria for CC were thickened subepithelial collagen layer ≥10 μm and increased lymphoplasmacytic cell infiltrate in the lamina propria. The histopathological criteria for LC were ≥20 lymphocytes in the epithelial layer per 100 epithelial cells without thickened collagen. Both aCC and LC were defined as more than 3 bowel movements per day or at least 1 watery bowel movement per day during a 1-week registration period. Clinical remission (tCC) was defined as less than three bowel movements/day and no watery bowel movement during a 1-week registration (only CC patients available: tCC, paired to a subset of aCC patients) [38]. A diagnosis of rCC was reached if patients did not achieve remission after 9 mg of budesonide treatment for 12 weeks. For patients with MC and HC, biopsies were taken from the descending colon. From patients with UC, biopsies were taken from the sigmoid colon or rectum from the inflamed part, approximately 5 cm distal to the border of inflammation. Biopsies from IBS were taken from the sigmoid colon. After collection, biopsies from all groups were preserved in 4% paraformaldehyde for histology analysis and immunofluorescent staining.

### 2.3. Immunofluorescence

Colonic biopsies from patients and controls were embedded in paraffin, sectioned at 5 μm and incubated at 60 °C for 3 h. Deparaffinization and rehydration were performed by routine procedure, where slides were subsequently incubated in 99.5%, 95%, and 70% ethanol and in distilled H_2_O [30].

For EGC staining, antigen retrieval was performed in citrate buffer (10 mM tri-sodium citrate dihydrate in H_2_O, pH 6, 0.5% Tween 20) using a pressure cooker (Aptum, Retriever 2100, Prestige medical LTD. East House, Blackburn, UK). After cooling down to room temperature, the sections were permeabilized in PBS-0.1% Triton 100X for 10 min, followed by blocking with 1% BSA in PBS-0.5% Tween and 300 mM glycine for 30 min. Sections were stained for rabbit-anti-GFAP (1:500, Dako Cytomation, Glostrup, Denmark) or rabbit-anti-S100β (1:200, Abcam, Amsterdam, The Netherlands) to detect EGC, followed by Alexa Fluor 546-conjugated goat-anti-rabbit secondary antibody (1:1000; Invitrogen, Groningen, The Netherlands) and mounted with Prolong^®^ Gold DAPI (Thermo Fisher, Stockholm, Sweden). For mast cells, the staining was, after deparaffinization, performed with a mouse-anti-mast cell tryptase antibody (1:200, Santa Cruz, Heidelberg, Germany) followed by Alexa fluor 546-conjugated goat-anti-mouse secondary antibody (1:1000; Invitrogen, The Netherlands), and the wheat germ agglutinin (WGA) 633 was used to identify cell membranes (1:500, Thermo Fisher, Stockholm, Sweden). Slides were rinsed and mounted with Prolong^®^ Glass antifade with DAPI (Thermo Fisher, Stockholm, Sweden). Secondary antibody specificity was confirmed by omitting the primary antibodies in all staining procedures.

### 2.4. Quantification of EGC and Mast Cells

For quantification, images at objective 63X were acquired using an inverted fluorescence Leica DMi8 microscope (Leica Microsystems CMS, Wetzlar, Germany) equipped with Hamamatsu ORCA Flash 4 (SCMOS) camera (Hamamatsu, Hamamatsu City, Japan) and Leica software version 3.9 (Leica Microsystems CMS, Wetzlar, Germany). Five random images per biopsy section were blindly manually quantified using the open-source platform Fiji package for ImageJ software version 1.53t (National institutes of Health, Bethesda, MD, USA) [39]. Results are presented as cell density, defined as the mean number of positive cells per field of view (mm^2^).

### 2.5. Analysis of Mast Cell Degranulation

To assess the granule distribution of tryptase, eight images of individual mast cells were captured using an inverted confocal Leica Stellaris 5 microscope (Leica Microsystems CMS) fitted with a 63× oil immersion objective (numerical aperture 1.4). Image processing was performed using the Fiji package for ImageJ version 1.53t (National institutes of Health, USA), and quantitative analysis was performed using CellProfiler version 2.2.0 (Broad institute Inc., Cambridge, MA, USA), following previously published methods [40]. Representative single-cell images were further handled in ImageJ version 1.53t (National institutes of Health, USA). In brief, raw confocal images were deconvolved using the PSF Generator and DeconvolutionLab2 plug-ins for ImageJ version 1.53t (National institutes of Health, USA) to correct out-of-focus blur originating from the specimen. A high-pass filter was subsequently applied using the GIMP version 2.10.36 open-source image processing software. Image quantification was performed on raw images, as follows: cell boundaries were defined and segmented based on nuclear staining with DAPI in combination with the WGA signal. Granules positive for tryptase were detected using Otsu thresholding and categorized as either intracellular or extracellular to evaluate degranulation. For each individual cell, the total intracellular signal and the extracellular signal in the surrounding area were measured, and mean intensity values were calculated across all analyzed cells. Results are reported as mean intensity/cell.

### 2.6. Staining and Quantification of Tight Junctions

Staining for the tight junction proteins claudin-1 and tricellulin was performed for the aCC and tCC groups to identify potential differences in tight junction expression after budesonide treatment. Formalin-fixed paraffin-embedded biopsy sections were deparaffinized in xylene and rehydrated through ethanol series as described in paragraph 2.4. Antigen retrieval was performed in citrate buffer (10 mM, pH 6.0) by microwaving for 10 min. After cooling, sections were rinsed in PBS, permeabilized in Triton 100X for 5 min and blocked with background Sniper (Biocare medical, Pacheco, CA, USA). Sections were stained overnight at 4 °C for tricellulin (rabbit; 1:100, Invitrogen) and claudin-1 (mouse; 1:100, Invitrogen, The Netherlands), diluted in glycine buffer (0.3 M glycine in PBS-0.1% Tween) containing 3% BSA (Sigma-Aldrich, St. Louis, MO, USA). Following washing, sections were incubated for 1 h at room temperature with secondary antibodies (donkey anti-rabbit 594; 1:500 and donkey anti-mouse 488, Invitrogen, The Netherlands). Slides were rinsed, nuclei were counterstained with one NucBlue™ Fixed cell stain (Invitrogen, The Netherlands) for 10 min and mounted with Prolong^®^ Glass antifade (Invitrogen, The Netherlands). Sections were monitored in a blind fashion for both tricellulin and claudin-1 using an inverted confocal Leica Stellaris 5 microscope (Leica Microsystems CMS, Wetzlar, Germany) fitted with a 63× oil immersion objective (numerical aperture 1.4). Five pictures per sample were taken. Epithelial tricellulin and claudin-1 were quantified using a 20-pixel-wide line that was drawn along the epithelial linings in each picture and evaluated in ImageJ version 1.53t (National institutes of Health, USA) [41]. The average intensity of the selected area was assessed and normalized for different lengths of the epithelial border. A mean value of the five images was calculated and presented in the results.

### 2.7. Analysis of Public RNA-Sequencing Datasets

Candidate EGC-related gene expression was analyzed from our datasets previously made available in GEO datasets GSE159010 (CC) [42] and GSE245764 (LC) [11]. Raw count data was normalized using DESeq2 (log2-transformed read counts), and differential gene expression was calculated using the Mann–Whitney (unpaired) or Wilcoxon (paired aCC-tCC) tests with a significance threshold of *p* < 0.05 (all performed in R Bioconductor software version 2026.08.2).

### 2.8. Statistical Analysis

Immunofluorescent data were statistically analyzed using GraphPad Prism version 10.0.2 (GraphPad Software Inc., Boston, MA, USA). The distribution of data was tested for normality using the Shapiro–Wilk test. Corresponding W-statistics and *p*-values are provided in Supplementary Table S1. Depending on data distribution, comparisons between more than two groups were performed using either one-way ANOVA with Tukey’s multiple comparisons test for normally distributed datasets (EGC^GFAP+^, mast cell density and mast cell degranulation) or the Kruskal–Wallis test with Dunn’s multiple comparisons test for non-normally distributed datasets (EGC^S100β+^, claudin-1 and tricellulin). Differences between paired samples (15 pairs of aCC and tCC) were analyzed using either a paired *t*-test or the Wilcoxon matched-pairs signed-rank test, depending on data distribution. RNA-sequencing datasets were analyzed in R. Data are expressed as mean ± SEM or median (interquartile range), and differences were considered significant for *p* < 0.05.

## 3. Results

### 3.1. EGC^GFAP+^ Cell Density Increases During Active CC, but Not During Active LC, and Is Reduced When Clinical Remission Is Reached Following Budesonide Treatment

**Figure 2:**
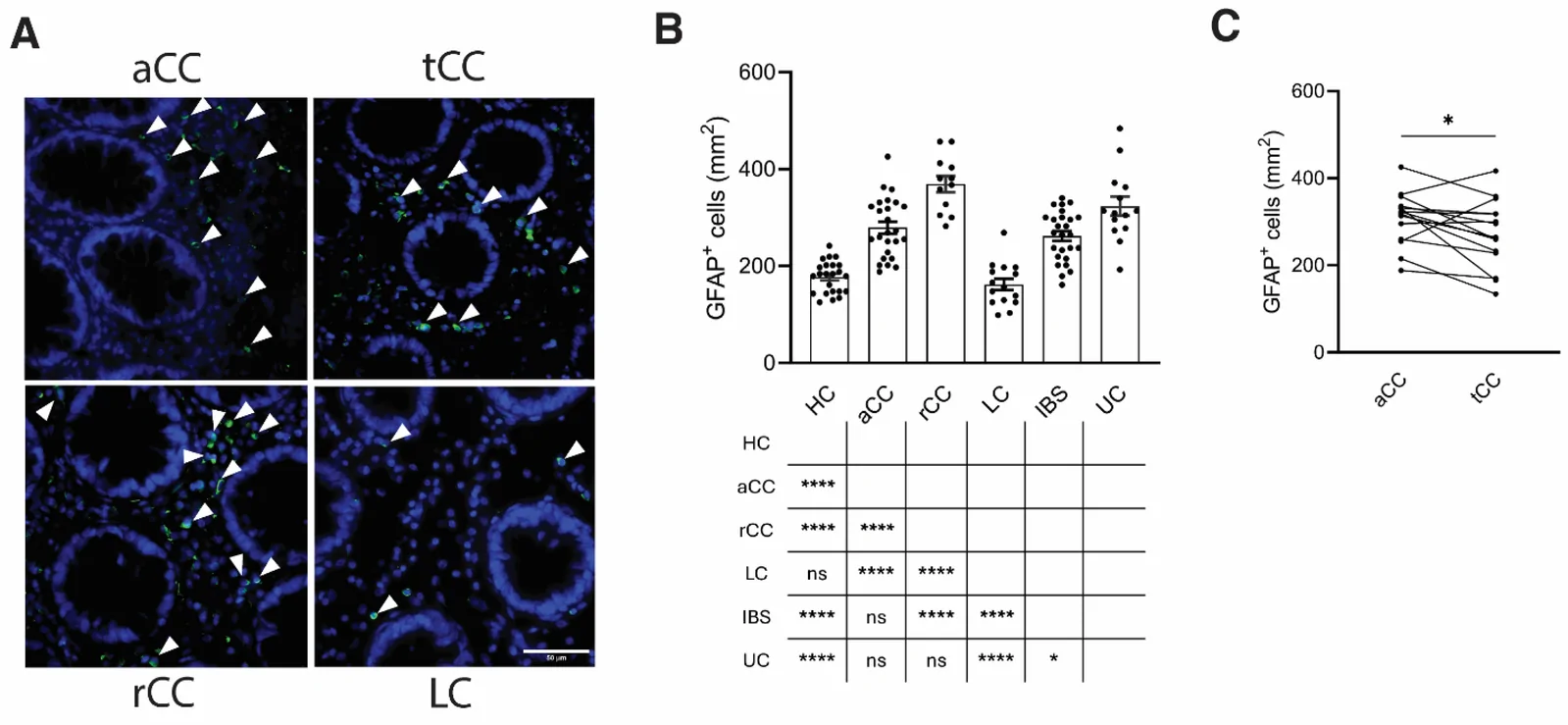
Glial fibrillary acidic protein (GFAP) expressing enteric glial cells (EGC) in colonic biopsies from microscopic colitis patients and controls. (**A**) Representative images of EGC^GFAP+^ (green) cells in active collagenous colitis (aCC), budesonide-treated CC achieving clinical remission (tCC), budesonide-refractory CC (rCC), and active lymphocytic colitis (LC). Scale bars show 50 μm. Arrowheads indicate EGC^GFAP+^. (**B**) EGC^GFAP+^ density in healthy controls (HC) and microscopic colitis cohorts (aCC, rCC, LC), and in irritable bowel syndrome (IBS) with diarrhea, and ulcerative colitis (UC), *n* = 12–26 patients/group. Statistical differences are indicated under the graph. (**C**) EGC^GFAP+^ density in paired samples (*n* = 15) from CC patients before/active (aCC) and after responsive budesonide treatment (tCC). Data were normally distributed and analyzed using one-way ANOVA with adjustments for multiple comparisons. Paired samples were analyzed using paired *t*-test. Data are expressed as mean ± SEM, * *p* < 0.05 and **** *p* < 0.0001, ns = non-significant.

The total number of EGC^GFAP+^ was quantified in immunofluorescence-stained sections from patients with MC, HC, and disease control groups including IBS and UC. The number of EGC^GFAP+^ per mm^2^ increased in active CC and aCC compared to active LC mucosa (*p* < 0.0001) (Figure 2A,B). However, the highest levels were observed in those CC patients who were treated with budesonide but remained unresponsive, rCC, with more EGC^GFAP+^ than in both aCC and LC (both *p* < 0.0001) (Figure 2A,B). Analysis of paired biopsies from a subset of CC patients obtained before and after budesonide treatment that induced clinical remission (aCC vs. tCC) showed a reduction in EGC^GFAP+^ following treatment (*p* < 0.05) (Figure 2A–C).

### 3.2. EGC^GFAP+^ Cell Density in CC Is Similar to Those Displayed in IBS and UC

Figure 2B shows EGC^GFAP+^ levels in MC subtypes compared with control groups. EGC^GFAP+^ was increased in aCC compared to HC (*p* < 0.0001). A similar increase compared to HC was observed in rCC (*p* < 0.0001), whereas no significant difference was seen between LC and HC. Importantly, no differences in EGC^GFAP+^ were observed between aCC and either IBS or UC, and no differences were observed for rCC and UC. In contrast, LC showed lower EGC^GFAP+^ compared to both IBS and UC (both *p* < 0.0001).

### 3.3. EGC^S100β+^ Cell Density Slightly Increases in Active CC but Is Not Affected by Budesonide Treatment

**Figure 3:**
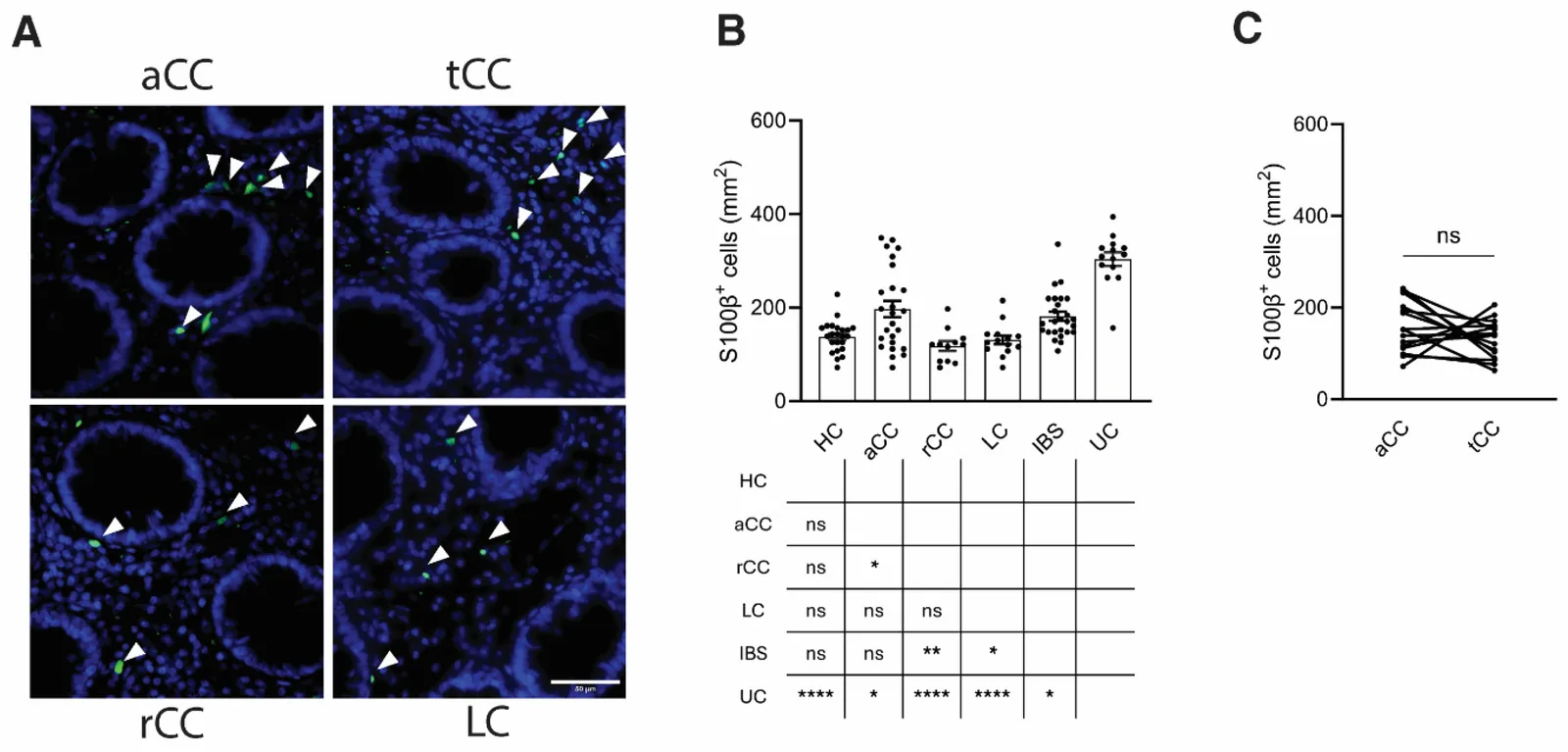
S100 calcium-binding protein β (S100β) expressing enteric glial cells (EGC) in colonic biopsies from microscopic colitis patients and controls. (**A**) Representative images of EGC^S100β+^ (green) staining in active collagenous colitis (aCC), budesonide-treated CC achieving clinical remission (tCC), budesonide-refractory CC (rCC), and active lymphocytic colitis (LC). Scale bars show 50 μm. Arrowheads indicate EGC^S100β+^. (**B**) EGC^S100β+^ density in healthy controls (HC) and microscopic colitis cohorts (aCC, rCC, LC), and in irritable bowel syndrome (IBS) with diarrhea, and ulcerative colitis (UC), *n* = 12–26 patients/group. Statistical differences are identified under the graph. (**C**) EGC^S100β+^ density in paired samples (*n* = 15) from CC patients before/active (aCC) and after responsive budesonide treatment (tCC). Data were not normally distributed and analyzed using Kruskal–Wallis test with adjustment for multiple comparisons. Paired samples were analyzed using Wilcoxon signed-rank test. Data are expressed as median ± interquartile range, * *p* < 0.05, ** *p* < 0.01, **** *p* < 0.0001, ns = non-significant.

EGC^S100β+^ were quantified in immunofluorescence-stained sections from MC subgroups and controls. EGC^S100β+^ numbers per mm^2^ were increased in aCC, although not statistically significant compared to LC samples (Figure 3A,B). In contrast to EGC^GFAP+^, EGC^S100β+^ were highest in aCC, with increased numbers compared to rCC (*p* < 0.05) (Figure 3A,B). However, no significant difference was observed between LC and other groups. Pairwise analysis of CC biopsies obtained before and after budesonide treatment showed no significant change in EGC^S100β+^ following treatment (Figure 3A–C).

### 3.4. EGC^S100β+^ Cell Density in CC Is Similar to That Present in IBS and Lower Compared to UC

Of the MC cohorts, only active aCC showed slightly elevated levels of EGC^S100β+^ compared to HC (Figure 3A,B). When compared to IBS, EGC^S100β+^ levels were similar to aCC and elevated compared to rCC (*p* < 0.01) and LC (*p* < 0.05) (Figure 3A,B). Compared to UC, all other patient groups displayed lower EGC^S100β+^ numbers (*p* < 0.0001) (Figure 3B).

### 3.5. Mast Cell Numbers Increase in Active CC, but Not in LC

**Figure 4:**
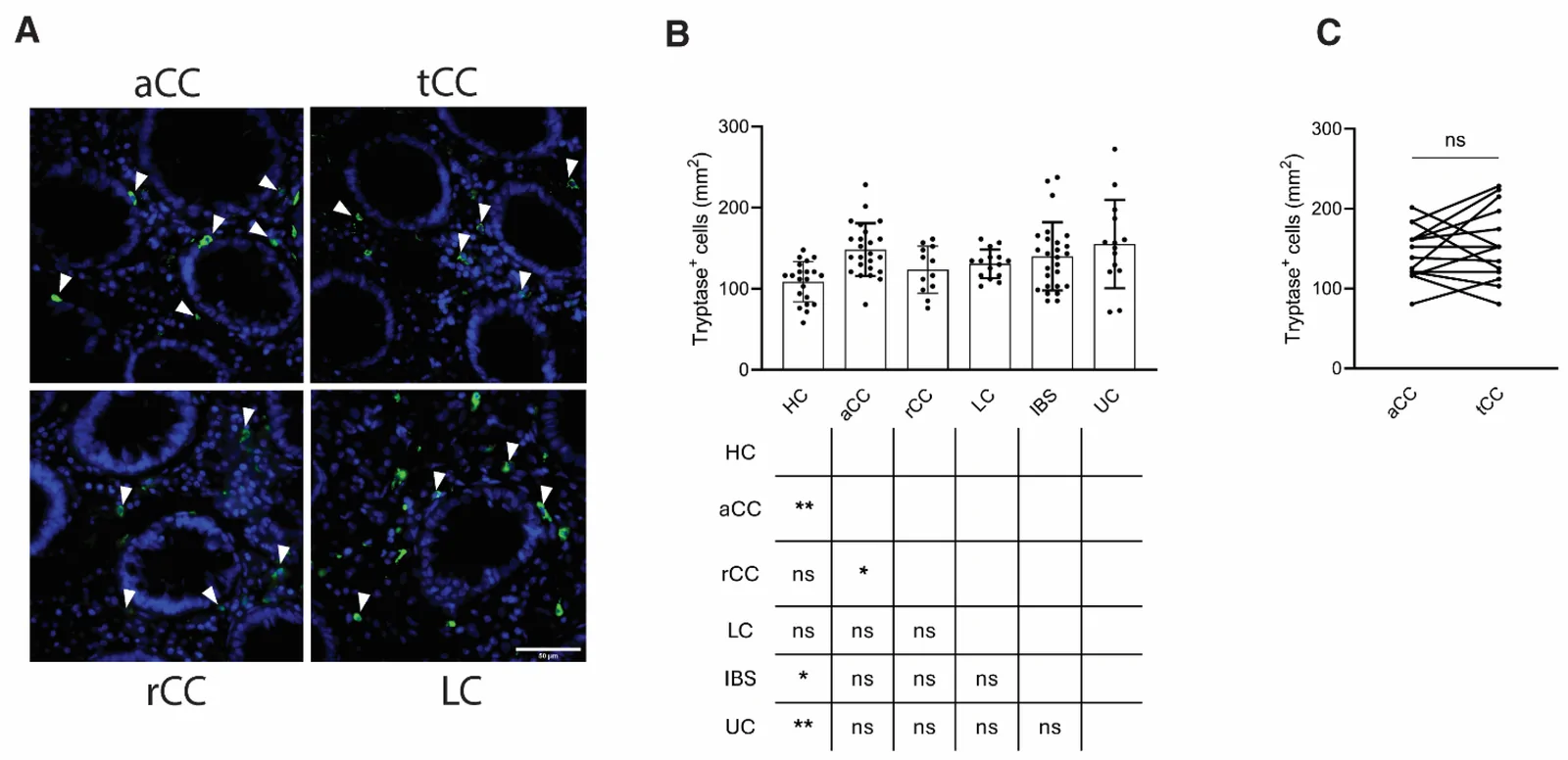
Tryptase-expressing mast cells in colonic biopsies from microscopic colitis patients and controls. (**A**) Representative images of mast cell^tryptase+^ (green) in active collagenous colitis (aCC), budesonide-treated CC achieving clinical remission (tCC), budesonide-refractory CC (rCC), and active lymphocytic colitis (LC). Scale bars show 50 μm. Arrowheads indicate mast cell^tryptase+^. (**B**) Mast cell density in healthy controls (HC) and microscopic colitis cohorts (aCC, rCC, LC), and in irritable bowel syndrome (IBS) with diarrhea, and ulcerative colitis (UC), *n* = 12–26 patients/group. Statistical differences are identified under the graph. Statistical differences are identified under the graph. (**C**) Mast cell density in paired samples (*n* = 15) from CC patients before/active (aCC) and after responsive budesonide treatment (tCC). Data were normally distributed and analyzed using one-way ANOVA with adjustment for multiple comparisons. Paired samples were analyzed using paired *t*-test. Data are expressed as mean ± SEM, * *p* < 0.05, ** *p* < 0.01, ns = non-significant.

The total number of mast cells was quantified as tryptase^+^ cells in immunofluorescence-labelled sections from MC subgroups and controls. Among the different MC groups, mast cell^tryptase+^ numbers were higher in aCC compared to rCC (*p* < 0.05). LC was not found to differ from any group (Figure 4A,B). Paired analysis revealed no significant difference in mast cell numbers before (aCC) and after budesonide treatment (tCC) (Figure 4A–C).

### 3.6. The Increase in aCC Mast Cell Numbers Is Similar to That of IBS and UC Samples

Compared with HC, mast cell numbers were increased in aCC (*p* < 0.01), IBS (*p* < 0.05) and UC (*p* < 0.01) (Figure 4B). No significant differences were observed between the IBS and/or UC groups with any of the MC subtypes, indicating comparable mast cell levels across MC, IBS and UC (Figure 4B).

### 3.7. Decreased Mast Cell Degranulation in MC After Budesonide Treatment

**Figure 5:**
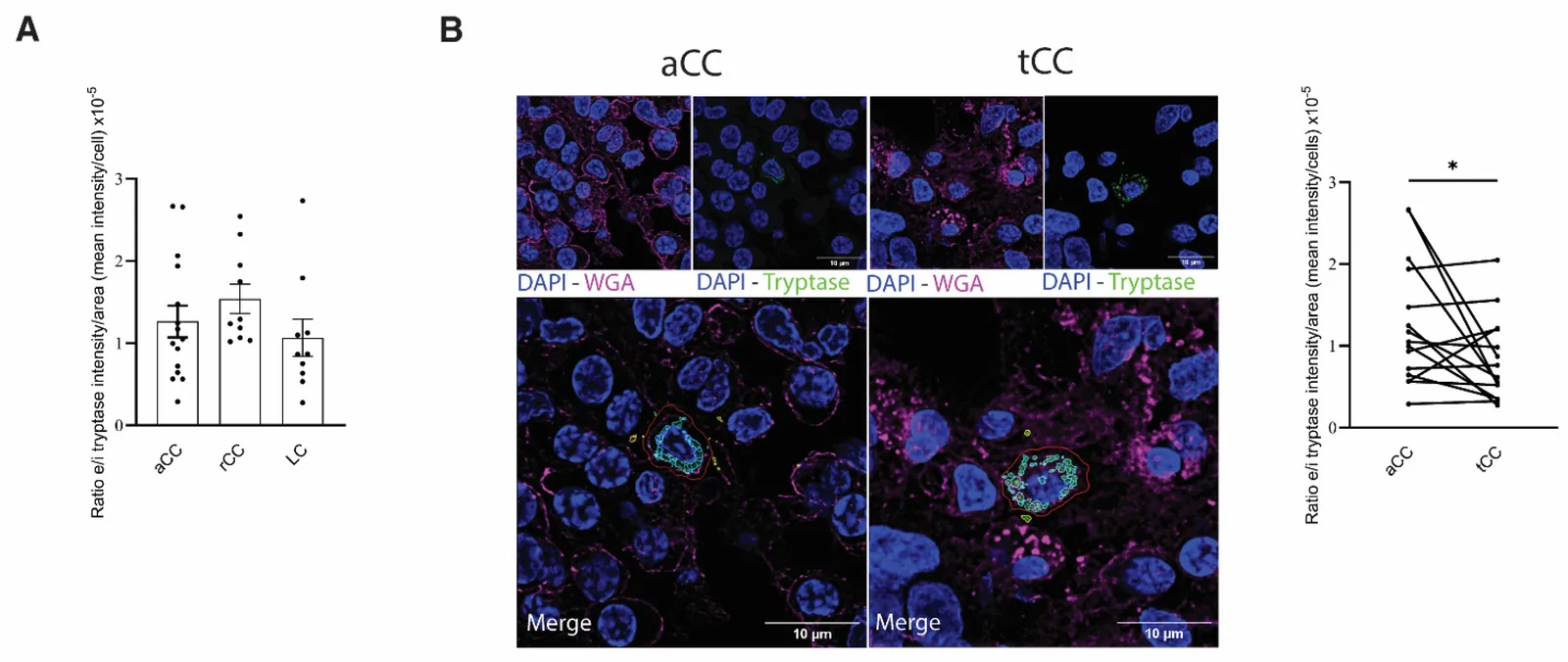
Mast cell degranulation measured as the ratio of extracellular and intracellular tryptase intensity in biopsies from patients with microscopic colitis. (**A**) Ratio of extracellular and intracellular tryptase intensity per area (mean intensity/cell) in patients with aCC, budesonide-refractory CC (rCC), and lymphocytic colitis (LC). (**B**) Representative images and ratio of extracellular and intracellular tryptase intensity per area (mean intensity/cell) in paired patients before and after treatment of an individual mast cell before (aCC) and following budesonide treatment (tCC). On the merged images, cell boundaries are outlined in red; intracellular (i) and extracellular (e) granules are enclosed respectively by a light blue and a yellow line. Scale bar shows 10 μm. Data were normally distributed and analyzed using one-way ANOVA with adjustment for multiple comparisons. Paired samples were analyzed using paired *t*-test. Data are expressed as mean ± SEM, * *p* < 0.05.

To further evaluate mast cell changes, in situ analyses of mast cell degranulation were performed by analyzing the extracellular to intracellular tryptase ratio in eight randomly selected mast cells per sample. This analysis demonstrated no significant differences in the ratios of extracellular to intracellular tryptase intensity between any of the MC subtypes (Figure 5A). However, a reduction in the ratio, reflecting decreased mast cell degranulation, could be observed after budesonide treatment (tCC) compared with baseline (aCC), *p* < 0.05 (Figure 5B). Representative images of mast cell degranulation in aCC and tCC are shown in Figure 5B.

### 3.8. Similar Expression of Tight Junction Proteins Before and After Budesonide Treatment

**Figure 6:**
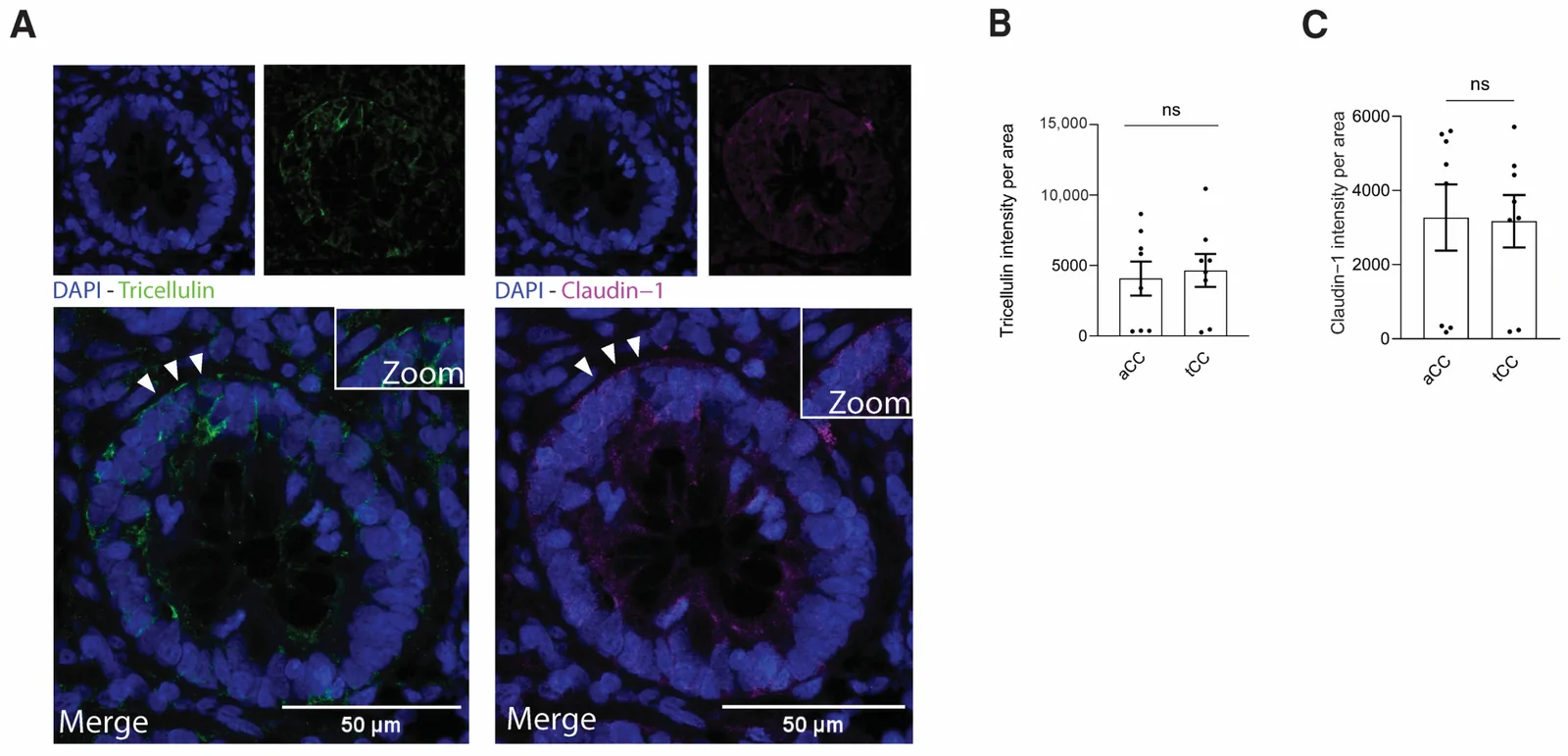
Expression of tight junction proteins in colonic biopsies from patients with active collagenous colitis (aCC) and in the same patients after budesonide treatment (tCC). Biopsies were stained for tricellulin and claudin-1, and their epithelial intensity was quantified using Fiji. (**A**) Photo showing a representative staining of tricellulin and claudin-1. On the merge image is a close-up of the area of interest. (**B**) Intensity of tricellulin per area and (**C**) intensity of claudin-1 per area (AU). Data were non-normally distributed and analyzed using Wilcoxon signed-rank test. Data are expressed as median (interquartile range). ns = non-significant.

To investigate barrier function after budesonide treatment, tight junctions were quantified in immunofluorescence for tricellulin and claudin-1. Representative images of tight junction proteins stained for tricellulin and claudin-1 with close-up views of the areas of interest are shown in Figure 6A. Staining of tricellulin (Figure 6A) and claudin-1 (Figure 6B) showed no significant differences between baseline (aCC) and after budesonide treatment (tCC).

### 3.9. Increased Expression of Immune- and Neuroglial-Related Genes in CC

**Figure 7:**
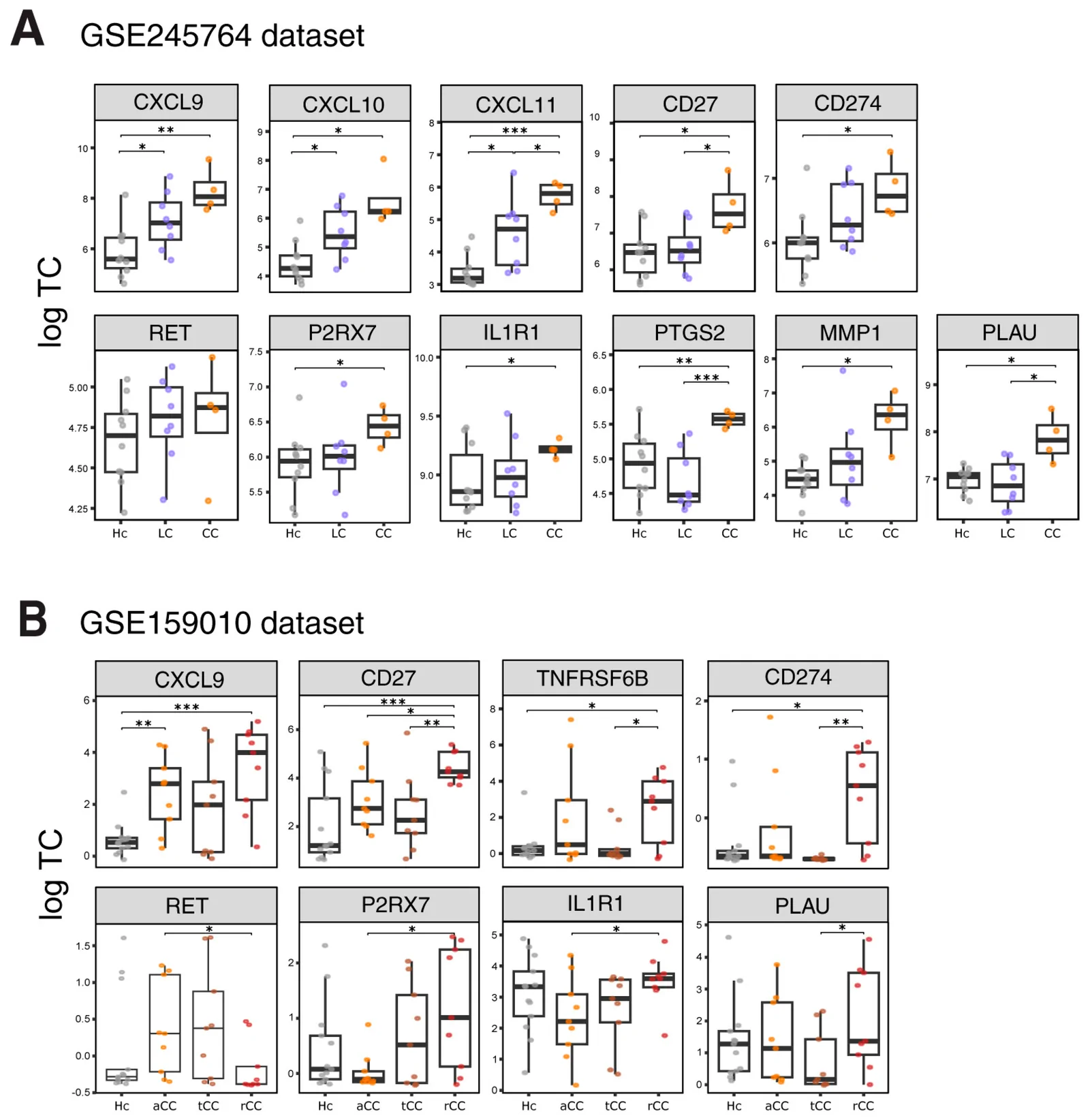
Immune and enteric glial cell-related genes are overexpressed in collagenous colitis. (**A**,**B**) Normalized log2-transformed transcript counts (log TC) in gene expression of candidate genes in (**A**) healthy controls (HC, grey, *n* = 10), lymphocytic colitis (LC, purple, *n* = 8) and collagenous colitis (CC, orange, *n* = 4) colonic mucosa from GSE245764 RNA-sequencing dataset; and in (**B**) healthy controls (HC, grey, *n* = 13), active CC (aCC, orange, *n* = 9), budesonide-responsive treated CC (tCC, brown, *n* = 9), and budesonide-refractory CC (rCC, red, *n* = 9) from GSE159010 RNA-sequencing dataset. Data is shown as median with IQR. Statistical differences are shown as * *p* < 0.05, ** *p* < 0.01, and *** *p* < 0.001. Genes that are not displayed in both cohorts did not reach the quality level for analysis in the cohort where that gene was not represented.

As EGC numbers, particularly those expressing GFAP, seemed to distinguish CC from LC, gene expression of key immune and neuroglial pathways was further explored using our previously published RNA-sequencing data from colonic CC and LC mucosa [11,42]. As previously suggested [11], MC overexpresses immune-related genes such as chemokines *CXCL9-11* in an increasing gradient, with CC displaying further overexpression than LC mucosa (Figure 7A). A similar trend in gene expression was observed for genes involved in T- and B-cell activation, *CD27*, and immune checkpoint ligand *CD274*/PD-L1 (Figure 7A). In addition, the ATP-gated ligand ion channel *P2RX7*, key for glial reactivity, increases in aCC. This, together with a similar increase in the expression of downstream genes in NLRP3 inflammasome (*IL1R1*) and prostaglandin (*PTGS2*) signaling pathways, may reflect an inflammatory tissue environment involving pathways with reported neuroglial relevance, particularly in CC (Figure 7A). Differential expression of genes associated with extracellular matrix remodeling, *PLAU* and *MMP1*, was also increased in CC (Figure 7A).

In the CC cohorts, the increase in *CXCL9*, *CD27* and *CD274*/PD-L1, in addition to *TNFRSF6B*, in active CC was confirmed, with rCC displaying further overexpression compared to the untreated aCC group (Figure 7B). In patients responding to budesonide (tCC), the expression of these genes was partially corrected to normal levels (Figure 7B). Neuroglial gene *P2RX7* increased only in rCC mucosa in this cohort, suggesting that these immune- and neuroglial-related transcriptional alterations may be more pronounced in refractory disease.

## 4. Discussion

The pathophysiology of MC remains poorly understood and, in this context, our study provides new insights into the involvement of EGC and mast cells. To our knowledge, this is the first study investigating EGC alterations in MC. We identified an increase in EGC expressing GFAP or S100β in CC mucosa compared to that of LC patients, suggesting distinct glial states across MC subtypes. Importantly, EGC^GFAP+^ numbers were restored after budesonide treatment. Still, EGC^S100β+^ cell numbers in rCC cases remained as high as those observed in UC mucosa (even higher than untreated active CC), which may reflect a persistent reactive EGC phenotype in refractory disease. Therefore, the distinct expression patterns observed for the two EGC markers, GFAP and S100β, suggest that they may reflect different aspects of EGC biology. Indeed, EGC comprise a heterogeneous population of cells with distinct phenotypic and functional properties that appear to be shaped by local cues within the gut microenvironment, including inflammation, microbial signals, and epithelial interactions [28,43]. Although mucosal EGC express both GFAP and S100β [25,44], the relative expression of these markers may vary depending on EGC subtype and microenvironment. Previous studies [45,46] have also reported incomplete overlap between GFAP and S100β expression [44], highlighting the heterogeneity of the EGC network and the need for additional methodological approaches to better define EGC subpopulations according to properties [43]. Therefore, the divergent patterns observed in our study may reflect distinct EGC phenotypes or differences in EGC density.

The increased density of EGC^GFAP+^ observed in patients with CC may indicate a reactive glial phenotype that has been described in other gastrointestinal disorders, including CD, UC [47] and IBS [48]. The reduction in EGC^GFAP+^ following budesonide treatment may suggest attenuation of this state. Although the mechanisms underlying this response remain unclear, accumulating evidence indicates that EGC are responsive to glucocorticoid signaling and may act as mediators between inflammatory cells and glucocorticoids [49]. Schneider et al. [49] demonstrated that elevated glucocorticoid levels drive the emergence of an inflammatory EGC subset characterized by activation of pro-inflammatory and pro-apoptotic pathways. In their mouse model of dextran sodium sulfate-induced colitis exposed to prolonged psychological stress, dexamethasone alone was sufficient to induce this stress-associated EGC phenotype, supporting the concept that EGC act as a relay between glucocorticoid signaling and inflammatory responses in the gut. Whether similar mechanisms contribute to the changes observed following budesonide treatment in MC remains to be determined.

EGC^GFAP+^ have been implicated in tissue repair and regeneration following injury as an important source of WNT ligands required for intestinal stem cell renewal [50]. Therefore, their increased presence in active CC mucosa might contribute to crypt hyperproliferation [42]. S100β has traditionally been used as a marker of EGC under physiological conditions [51], and transcriptomic studies indicate that most colonic EGC in the healthy adult intestine express S100β together with PLP1, whereas GFAP expression is absent or restricted to a small subset of cells [52,53]. Cirillo et al. [44] further suggested an active role for S100β in UC by demonstrating its involvement in nitric oxide production and mucosal immune responses. Of note, increased nitric oxide production has also been reported in both CC and LC [54].

Our findings support the hypothesis that an increased density of EGC^GFAP+^ represents a reactive EGC phenotype that may be linked to tissue repair and neuroimmune adaptation. This interpretation is in line with our previous findings in both UC and CD, where alterations in EGC marker expression were associated with more pronounced inflammatory conditions [14,30]. In contrast, the differing EGC^S100β+^ pattern observed in MC may reflect other aspects of glial biology that are less directly associated with inflammatory activity and may instead relate to differences in EGC subtype composition or functional state.

EGC and mast cells are known to engage in neuroimmune communication in gastrointestinal disorders. In CD, EGC acquire antigen-presenting properties and promote inflammation through the recruitment of immune cells [55]. In IBS, mast cells, which are recognized regulators of gastrointestinal motor activity [56], showed disrupted interactions with EGC in the colonic mucosa [24]. Increased mast cell numbers have previously been reported in patients with chronic diarrhea [57] and in IBS [58]. In the present study, mast cell numbers were increased only in aCC compared with HC, while Chi et al. [31] found increased mast cell counts and elevated extracellular tryptase levels in the lamina propria of both CC and LC patients compared with HC. Mast cells have therefore previously been implicated in the pathophysiology of MC. Although no significant differences in mast cell number were observed following budesonide treatment in our cohort, previous studies suggest that immune cell activation and mediator release may contribute more substantially to epithelial barrier dysfunction than immune cell numbers alone [59,60]. This may explain our finding that reduced mast cell degranulation follows treatment despite unchanged mast cell counts. The decrease in the extracellular-to-intracellular tryptase ratio following budesonide treatment indicates reduced mast cell degranulation and mediator release. Taken together, our findings support that mast cell degranulation rather than mast cell abundance may be associated with treatment responsiveness in CC. The parallel alterations observed in EGC markers and mast cell degranulation support the involvement of multiple neuroimmune components in CC but do not establish a direct interaction between EGC and mast cells. Future spatial and functional studies are required to determine whether these cell populations communicate directly in MC.

Beyond neuroimmune alterations, epithelial barrier dysfunction is a recognized feature of MC and has been proposed to contribute to the development of chronic diarrhea. In this context, tight junction proteins claudin-1 and tricellulin were investigated as potential regulators of barrier integrity. Tricellulin was of particular interest given its emerging role in epithelial barrier regulation, and our recent finding demonstrated altered tricellulin expression following a fermented oat-based intervention in IBS [15]. In the present study, however, we did not observe any changes in the expression of either protein following budesonide treatment. Although evidence specifically addressing claudin-1 expression in MC is limited, our findings of unchanged claudin-1 expression in CC and LC are consistent with findings reported in a previous study [61]. To our knowledge, no studies have previously investigated tricellulin expression in MC. Our results suggest that claudin-1 and tricellulin are unlikely to contribute substantially to the effects of budesonide in CC. However, as only two tight junction proteins were evaluated using immunofluorescence, alterations in other epithelial components, such as other claudins and ZO-1 as previously described [10,11], cannot be excluded and should be explored in future studies using complementary approaches.

To further explore mechanisms underlying the observed EGC alterations in MC, revision of RNA-sequencing data identified increased gene expression of immune (*CXCL9-11*, *CD27*, *CD274*) and neuroglial pathways (*P2RX7* and downstream genes *IL1R1* and *PTGS2*) in CC compared to LC [62]. Notably, CXCL10 has been linked to EGC activation under inflammatory conditions [63], whereas CXCL9 has been associated with inflammatory glial responses, including microglia within the central nervous system [64]. Increased expression of these chemokines may therefore reflect an inflammatory microenvironment capable of influencing EGC responses in MC. Interestingly, elevated *CXCL9* and *P2RX7* expression in rCC compared to aCC mucosa may be consistent with the persistently increased EGC^GFAP+^ population observed in refractory patients. Together, these findings may reflect ongoing EGC-associated immune activity in refractory disease. In addition, differential RET expression was observed between aCC and rCC; however, the biological significance of this finding remains unclear and requires further investigation. Taken together, the transcriptomic findings indicate an inflammatory and potentially neuroglial mucosal environment in CC, particularly in patients with refractory disease. However, because these findings were derived from bulk RNA-sequencing, the cellular sources of the identified transcripts cannot be determined. Cell-specific transcriptomic or spatial approaches will therefore be required to establish whether these pathways are directly associated with EGC and to clarify their functional relevance to CC pathophysiology and treatment response.

## 5. Conclusions

In conclusion, to our knowledge, this study provides the first characterization of EGC in MC and identifies subtype-specific EGC alterations in CC and LC. We have identified an increase in EGC^GFAP+^, EGC^S100β+^ and mast cell degranulation in CC. Several of these observed alterations resembled patterns previously reported for IBS with diarrhea and UC, suggesting shared neuroimmune mechanisms between CC and these disorders, while others appeared to be subtype-specific within MC. Importantly, EGC^GFAP+^ cell density was modulated by budesonide treatment in CC patients. On the other hand, LC displayed similar cell densities and mast cell degranulation as non-inflamed controls, indicating fewer neuroimmune alterations in the specific EGC and mast cell parameters investigated and suggesting distinct pathogenic mechanisms compared to CC. While no treatment-related changes were observed in the tight junction proteins claudin-1 and tricellulin, our data emphasize the importance of neuroimmune alterations in MC. In addition, revision of gene expression of chemokines and pathways related to EGC biology suggests altered microenvironments that could affect EGC signaling in CC, especially in those patients who fail to respond to budesonide. Transcriptomic analyses revealed alterations in immune signaling, extracellular matrix remodeling and neuroglial pathways. Therefore, our work provides a framework for future EGC-oriented studies to characterize their contribution to CC and to budesonide response.

## Figures and Tables

**Table 1:** Clinical and demographic characteristics of the 52 patients with microscopic colitis included in the study.

Diagnosis	Sex	Age at Diagnosis (Years)	Disease Duration at Endoscopy (Years)	Smoking Status	Mean Stools/Day	Mean Watery Stools/Day
CC	F	72	3	No	10	10
CC	F	55	6	No	7	7
CC	F	65	8	No	8	8
CC	F	37	7	No	6	6
CC	F	72	5	No	7	7
CC	F	51	1	No	20	20
CC	M	37	4	Yes	8	8
CC	F	71	0	No	3	2
CC	F	53	3	No	5	5
CC	F	79	0	No	5	5
CC	F	67	6	No	10	9
CC	F	69	1	Yes	8	8
CC	F	38	3	Yes	4	4
CC	F	46	4	Yes	5	5
CC	F	47	14	No	8	8
CC	F	25	1	Unknown	7	7
CC	F	68	1	Yes	6	6
CC	M	70	2	Unknown	7	7
CC	F	30	11	Unknown	7	7
CC	F	34	4	Unknown	7	7
CC	M	54	1	Unknown	5	5
CC	F	48	1	Yes	6	6
CC	F	69	7	No	4	3
CC	M	33	2	Unknown	5	5
CC	F	78	8	Yes	8	8
rCC	M	59	5	Yes	10	6
rCC	F	65	1	Yes	15	15
rCC	F	75	8	Unknown	6	5
rCC	F	57	7	No	8	8
rCC	F	51	3	Unknown	6	5
rCC	F	52	10	Unknown	6	6
rCC	F	55	4	Unknown	8	8
rCC	F	63	6	Yes	4	4
rCC	F	35	7	Yes	6	6
rCC	F	51	1	No	9	8
rCC	F	30	13	Yes	10	10
rCC	F	25	1	Unknown	15	15
LC	M	57	2	No	10	10
LC	F	62	6	Unknown	4	4
LC	F	58	0	No	5	4
LC	F	39	2	No	7	7
LC	M	53	0	No	4	1
LC	F	68	0	No	5	1
LC	F	63	0	No	3	2
LC	M	53	0	Unknown	3	3
LC	F	67	3	No	7	7
LC	M	54	2	No	2	2
LC	F	19	0	No	6	6
LC	M	73	3	Yes	10	10
LC	F	80	1	No	4	3
LC	F	44	0	No	15	15
LC	F	51	1	No	6	6

NOTE: CC = collagenous colitis, F = female, LC = lymphocytic colitis, M = male, rCC = refractory collagenous colitis.

## Data Availability

The data that support the findings of this study are available on request from the corresponding author. The data are not publicly available due to privacy or ethical restrictions.

## Supplementary Materials

The following Supporting Information can be downloaded at: https://www.mdpi.com/article/10.3390/cells15191778/s1, Table S1: Assessment of normality using the Shapiro–Wilk test.
