# Supplementary material for: Differential Enteric Glial and Mast Cell Characteristics Distinguish Microscopic Colitis Subtypes Relative to Healthy and Disease Controls, with Treatment-Associated Reductions in Glial Reactivity and Mast Cell Degranulation in Collagenous Colitis
Source: Cells. 2026 Sep 29;15(19):1778. doi: 10.3390/cells15191778 (PMC13664090; doi:10.3390/cells15191778)
Supplement: Supplementary file 1 [file cells-15-01778-s001.zip › cells-4580613-supplementary.pdf]

Supplementary Table S1. Assessment of normality using the Shapiro-Wilk test.

Variable	Group	Shapiro-Wilk W-value	p-value
EGC^{GFAP}	HC	0.95	0.33
	aCC	0.95	0.31
	tCC	0.98	0.95
	rCC	0.95	0.63
	LC	0.94	0.43
	IBS	0.95	0.63
	UC	0.95	0.57
$EGC^{S100\beta}$	HC	0.97	0.69
	aCC	0.94	0.37
	tCC	0.97	0.89
	rCC	0.91	0.24
	LC	0.92	0.47
	IBS	0.91	*0.03*
	UC	0.89	0.08
Mast cell density	HC	0.95	0.39
	aCC	0.98	0.80
	tCC	0.95	0.52
	rCC	0.94	0.48
	LC	0.96	0.69
	IBS	0.91	0.10
	UC	0.96	0.67
Mast cell degranulation	aCC	0.91	0.11
	tCC	0.89	0.06
	rCC	0.85	0.06
	LC	0.85	0.06
Claudin-1	aCC	0.80	*0.01*
	tCC	0.88	0.16
Tricellulin	aCC	0.85	*0.04*
	tCC	0.92	0.43

NOTE: Normality was assessed using the Shapiro-Wilk test prior to statistical analysis. W-values and corresponding p-values are provided for each study variable and group. Italicized p-values indicate significant deviation from normality ($p<0.05$). The choice of parametric or non-parametric statistical methods was based on these assessments. Variables containing one or more groups with significant deviation from normality were analysed using non-parametric statistical tests, whereas variables fulfilling normality assumptions were analysed using parametric statistical tests. aCC= active collagenous colitis; EGC= enteric glial cells; GFAP= glial fibrillary acidic protein; HC=healthy controls; IBS=irritable bowel syndrome;

LC=lymphocytic colitis; rCC= refractory collagenous colitis; S100β= S100 calcium-binding protein B; tCC=treated collagenous colitis; UC=ulcerative colitis.
